# A phase 3, randomized, double-blind, multicenter, placebo-controlled study of S-588410, a five-peptide cancer vaccine as an adjuvant therapy after curative resection in patients with esophageal squamous cell carcinoma

**DOI:** 10.1007/s10388-024-01072-w

**Published:** 2024-07-11

**Authors:** Tomoki Makino, Hiroshi Miyata, Takushi Yasuda, Yuko Kitagawa, Kei Muro, Jae-Hyun Park, Tetsuro Hikichi, Takahiro Hasegawa, Kenji Igarashi, Motofumi Iguchi, Yasuhide Masaoka, Masahiko Yano, Yuichiro Doki

**Affiliations:** 1 Department of Surgery, Gastroenterological Surgery, Graduate School of Medicine, Osaka University, Osaka, Japan; 2 Department of Gastroenterological Surgery, Osaka International Cancer Institute, Osaka, Japan; 3 Department of Surgery, Kindai University Faculty of Medicine, 377-2, Ohno-Higashi, Osaka-Sayama, Osaka 589-8511 Japan; 4 Department of Surgery, Keio University School of Medicine, Tokyo, Japan; 5 Department of Clinical Oncology, Aichi Cancer Center Hospital, Nagoya, Aichi Japan; 6 OncoTherapy Science, Inc., Kawasaki, Kanagawa Japan; 7 Laboratory Department, Cancer Precision Medicine, Inc., Kawasaki, Kanagawa Japan; 8 Biostatistics Center, Shionogi & Co., Ltd., Osaka, Japan; 9 Project Management, Shionogi & Co., Ltd., Osaka, Japan; 10 Medical Affairs Department, Shionogi & Co., Ltd, Osaka, Japan; 11 Clinical Development, Shionogi & Co., Ltd, Osaka, Japan; 12 Present Address: Kyowakai Hospital, Osaka, Japan

**Keywords:** Esophageal squamous cell carcinoma, Adjuvant therapy, Cytotoxic T lymphocytes, Cancer peptide vaccine, S-588410

## Abstract

**Background:**

S-588410, a cancer peptide vaccine (CPV), comprises five HLA-A*24:02-restricted peptides from five cancer-testis antigens. In a phase 2 study, S-588410 was well-tolerated and exhibited antitumor efficacy in patients with urothelial cancer. Therefore, we aimed to evaluate the efficacy, immune response, and safety of S-588410 in patients with completely resected esophageal squamous cell carcinoma (ESCC).

**Methods:**

This phase 3 study involved patients with HLA-A*24:02-positive and lymph node metastasis-positive ESCC who received neoadjuvant therapy followed by curative resection. After randomization, patients were administered S-588410 and placebo (both emulsified with Montanide™ ISA 51VG) subcutaneously. The primary endpoint was relapse-free survival (RFS). The secondary endpoints were overall survival (OS), cytotoxic T-lymphocyte (CTL) induction, and safety. Statistical significance was tested using the one-sided weighted log-rank test with the Fleming–Harrington class of weights.

**Results:**

A total of 276 patients were randomized (*N* = 138/group). The median RFS was 84.3 and 84.1 weeks in the S-588410 and placebo groups, respectively (*P* = 0.8156), whereas the median OS was 236.3 weeks and not reached, respectively (*P* = 0.6533). CTL induction was observed in 132/134 (98.5%) patients who received S-588410 within 12 weeks. Injection site reactions (137/140 patients [97.9%]) were the most frequent treatment-emergent adverse events in the S-588410 group. Prolonged survival was observed in S-588410-treated patients with upper thoracic ESCC, grade 3 injection-site reactions, or high CTL intensity.

**Conclusions:**

S-588410 induced immune response and had acceptable safety but failed to reach the primary endpoint. A high CTL induction rate and intensity may be critical for prolonging survival during future CPV development.

**Supplementary Information:**

The online version contains supplementary material available at 10.1007/s10388-024-01072-w.

## Introduction

According to the Global Cancer Statistics 2020, esophageal cancer had 604,100 new cases and approximately 544,100 associated deaths, with the highest incidence and mortality documented in Eastern Asia [1]. By 2040, the incidence of esophageal cancer and the associated mortality is estimated to increase by 58% and 62% worldwide, respectively [2]. In contrast to Europe and the United States, Asia, including Japan, has a higher number of patients with esophageal squamous cell carcinoma (ESCC) than those with adenocarcinoma [2]; hence, treatment approaches and outcomes tend to differ across regions [3].

Surgery remains a major treatment strategy for locally advanced resectable esophageal cancers, and reports have suggested that the addition of preoperative or postoperative therapy to surgery could substantially improve patient survival [4, 5]. The Esophagus 2022 Guidelines for Diagnosis and Treatment of Carcinoma recommend nivolumab and pembrolizumab, both anti-programmed cell death (PD)-1 antibodies for patients with advanced or metastatic esophageal cancer. Moreover, adjuvant therapy with nivolumab has recently been approved for patients with stage II or III esophageal or gastroesophageal junction cancer who have received neoadjuvant chemotherapy and have residual pathological disease [6]. The efficacy of anti-PD-1 antibodies is associated with the presence of tumor-infiltrating lymphocytes (TILs) and the expression of programmed death ligand 1 (PD-L1) in tumors [7].

HLA-A*24:02 is one of the most frequently observed HLA-A alleles in Asian races with a particularly high frequency in Japanese individuals (approximately 60%) [8]. Previously, a cancer vaccine comprising three HLA-A*24:02-restricted peptides derived from three antigens, i.e., lymphocyte antigen-6 complex locus (LY6k, also known as upregulated lung cancer 10; URLC10), TTK protein kinase, and insulin-like growth factor-II mRNA binding protein-3 (IMP3, also known as KH domain-containing protein overexpressed in cancer 1; KOC1), was found to prolong survival and induce antigen-specific cytotoxic T lymphocytes (CTLs) in patients with ESCC with locally advanced, recurrent, or/and metastatic tumors who failed to respond to the standard therapy [9]. An investigator-initiated phase 2 study on three HLA-A*24:02-restricted peptides from URLC10, KOC1, and cell division cycle-associated protein 1 (CDCA1) demonstrated prolonged survival of patients with esophageal cancer presenting pathologically positive nodes and curatively resected after preoperative therapy [10]. Another cancer vaccine comprising two HLA-A*24:02-restricted peptides from DEP domain containing 1 (DEPDC1) and M-phase phosphoprotein 1 (MPHOSPH1) afforded prolonged survival in CTL-induced patients with advanced urothelial carcinoma of the bladder [11]. Initially, clinical trials were conducted on a vaccine containing DEPDC1- and MPHOSPH1-derived peptides for bladder cancer patients, who frequently express these two antigens. Meanwhile, a vaccine containing URLC10-, CDCA1-, and KOC1-derived peptides were used in clinical trials for esophageal and lung cancer patients, who frequently express these three antigens. However, since it was revealed that all five antigens were expressed in bladder and esophageal cancer [12, 13], a five-peptide vaccine, S-588410, was determined to be developed for a cancer vaccine against esophageal cancer. The development of cancer peptide vaccines to date has led to the recognition of the heterogeneity of cancer, in which antigen expression patterns differ from patient to patient and from cancer cell to cancer cell, and the immune response (CTL induction) to each peptide that differs from patient to patient [14, 15]. Therefore, vaccines containing peptides derived from multiple antigens are expected to be highly effective in a wider range of patients.

The S-588410 comprises five HLA-A*24:02-restricted peptides from cancer-testis antigens (URLC10, CDCA1, KOC1, DEPDC1, and MPHOSPH1) [16, 17]. It can increase the number of antigen-specific CTLs in the blood and promote CTL recruitment at the tumor site in the esophagus as TILs. Hence, S-588410 could be effective in patients with esophageal cancer [16]. Given that S-588410 was well-tolerated and exhibited antitumor efficacy in certain patients with urothelial cancer in a phase 2 study as a maintenance therapy, this vaccine may address the unmet need for adjuvant therapy in patients with cancer [17]. Accordingly, in the current randomized, placebo-controlled phase 3 study, we aimed to evaluate the efficacy, immune response, and safety of S-588410 as an adjuvant monotherapy for HLA-A*24:02-positive patients with completely resected ESCC.

## Methods

### Study design, vaccination, and ethics

This randomized, double-blind, multicenter, placebo-controlled study comprised four periods. The study duration was a minimum of 144 weeks and a maximum of approximately 5 years (96-week treatment period, 48-week post-treatment observation period, and follow-up period of up to 3 years after enrollment of the last participant). Eligible participants were enrolled and randomized at a ratio of 1:1 to the S-588410 or placebo group after HLA-A genotyping (Fig., Online Resource 1).

The participants were subcutaneously administered 1 mL of S-588410 emulsion, including 1 mg of each of the five peptides (URLC10, CDCA1, KOC1, DEPDC1, and MPHOSPH1) in Montanide™ ISA 51 VG (Seppic S.A., Paris, France), or 1 mL of placebo emulsion in Montanide™ ISA 51 VG into the inguinal, axillary, or cervical region once weekly for 12 weeks, followed by once every 2 weeks for 94 weeks.

This study (clinical trial number: UMIN000016954) was conducted across 55 centers in Japan in accordance with the International Council for Harmonization Good Clinical Practice and the guiding principles of the Declaration of Helsinki and was approved by the Institutional Review Board or Independent Ethics Committees and health authorities (Shionogi Ethical Committee: November 15, 2014). Written informed consent was obtained from all participants.

### Participants

The key inclusion criteria were as follows: HLA-A*24:02-positive, age ≥ 20 years, ESCC, neoadjuvant chemotherapy or chemoradiotherapy followed by curative resection classified as R0 according to the 7th edition of the Union for International Cancer Control (UICC), and histopathologically detected lymph node metastasis followed by lymph node dissection. The main exclusion criteria were serious concurrent disease (hepatic or renal disorder or cardiac, hematologic, respiratory, or metabolic disease) and laboratory test results indicating impaired blood, hepatic, or renal function within 28 days of enrollment. Other inclusion and exclusion criteria as well as methods for immunohistochemistry are presented in Online Resource 2.

### Outcomes

The primary efficacy endpoint was relapse-free survival (RFS), which was defined as the time interval from the date of randomization to the date of relapse or death by any cause, whichever occurred first, or to the date when no relapse was confirmed. The secondary endpoints were overall survival (OS), disease-free survival (DFS), CTL induction rate (investigated within 12 weeks after the initial dose), and safety. DFS was defined as the time interval from the date of randomization to the date of recurrence, death by any cause, or the date of diagnosis of a secondary cancer, whichever occurred first, or the date of confirmation of no disease. RFS and DFS were evaluated at the central judgement committee, established independently from the sponsor and the medical institutions conducted this study. The CTL induction rate was calculated as the proportion of participants who showed positive CTL induction towards at least one of the five peptides. Positive CTL induction was defined as an increase in CTL intensity at any point after the baseline (before dosing on the date of the first dose). Imaging examinations were performed every 12 weeks. Clinical tests were performed at 1 week, 6 weeks, and 12 weeks after randomization, and thereafter every 12 weeks. Adverse events (AEs) were classified by system organ class and preferred terms using the Medical Dictionary for Regulatory Activities (MedDRA) version 23.0. Assessment of the injection site, physical examination, ECOG PS, vital signs, electrocardiogram, laboratory tests, and assessment of AEs according to CTCAE version 4.03 were performed. Of the reported AEs, treatment-emergent adverse events, defined as AEs occurring after the first dose of S-588410 or placebo, were used for safety analyses.

### Enzyme-linked immunospot (ELISPOT) assay

For immune response monitoring, peripheral blood mononuclear cells (PBMCs) from participants in the S-588410 group were collected before vaccination (baseline) and at 8, 12, 32, 48, 96, and 108 weeks after vaccination and stored at − 80 °C until use. CTL induction, as an immunological response to each immunized peptide, was evaluated by counting spots in interferon-gamma-generating cells using an ELISPOT assay [9, 11]. After 2 weeks of in vitro PBMC stimulation with cognate peptides, cells were further incubated for 24 h in triplicate with TISI cells pulsed with each of the five peptides as stimulators. Subsequently, the number of peptide-specific spots was calculated based on the average of the triplicate measurements by subtracting the non-stimulated well (control) from the stimulated well containing the cognate peptide. According to a previously described scoring algorithm, the CTL response was classified into four grades (negative, 1+, 2+, and 3+) depending on the counts of peptide-specific spots [9].

### Statistics

The target number of participants was set to 135 for each group, given that a one-sided significance level of 5% and power of 80% were assumed to detect a difference in RFS between the groups, considering the small number of dropouts. The assumptions used to calculate the required sample size are presented in additional information in Online Resource 3. For the intention-to-treat (ITT) population, the RFS, OS, and DFS of the S-588410 group were compared with those of the placebo group using the weighted log-rank test with Fleming–Harrington weights for (ρ, γ) = (0, 0.5), stratified by the histopathological classification of lymph node metastasis (pN1, pN2, or pN3) at a one-sided significance level of 0.05. For the primary analysis plan, a fixed-sequence procedure with OS was implemented in case of a significant difference in RFS for multiplicity adjustment with these comparisons, whereby the type I error rate for this primary analysis was controlled at ≤ 5%. The survival curves for RFS, OS, and DFS were estimated using the Kaplan–Meier method for each treatment group, and the median RFS, OS, and DFS and the associated 90% confidence interval (CI) were estimated. Considering the secondary endpoint, the CTL induction rate was defined as the proportion of patients who showed in vitro CTL induction toward at least one of the five antigens in the analysis population. Details of the analysis plan for CTL induction are described in Online Resource 3. Statistical analyses were performed using SAS software (version 9.4; SAS Institute Inc., Cary, NC, USA).

## Results

A total of 276 participants were enrolled between May 2015 and March 2018, with 138 participants randomized to each of the S-588410 and placebo groups (ITT population) and followed up until March 2021. Two participants in the placebo group were mistreated with S-588410 (once each) and were included in the S-588410 group for safety analysis based on the study protocol (Fig., Online Resource 4). The two groups showed comparable features in terms of patient background (Table 1). Other baseline demographics, including clinical stage at diagnosis and antigen expression, were similar between the groups (Table, Online Resource 5; Fig., Online Resource 6).

**Table 1:** Characteristics of the participants in the intention-to-treat (ITT) population

	S-588410 *N* = 138	Placebo *N* = 138
Age (years), Mean ± standard deviation	65.0 ± 7.3	65.8 ± 8.2
Sex		
Male	109 (79.0)	114 (82.6)
Female	29 (21.0)	24 (17.4)
Tumor lesion		
Upper	19 (13.8)	22 (15.9)
Mid	67 (48.6)	66 (47.8)
Lower	52 (37.7)	50 (36.2)
Neoadjuvant therapy		
Chemotherapy	123 (89.1)	123 (89.1)
FP	66 (47.8)	59 (42.8)
DCF	56 (40.6)	62 (44.9)
FAP	1 (0.7)	2 (1.4)
Chemoradiotherapy	15 (10.9)	15 (10.9)
Lymphadenectomy		
2-field	32 (23.2)	27 (19.6)
3-field	106 (76.8)	111 (80.4)
ypTNM_T at enrollment		
T0	4 (2.9)	7 (5.1)
T1	31 (22.4)	39 (28.2)
T2	27 (19.6)	16 (11.6)
T3	72 (52.2)	76 (55.1)
T4	4 (2.8)	0
ypTNM_N at enrollment		
N0	5 (3.6)	3 (2.2)
N1	81 (58.7)	81 (58.7)
N2	40 (29.0)	41 (29.7)
N3	12 (8.7)	13 (9.4)
ypTNM_M at enrollment		
M0	116 (84.1)	116 (84.1)
M1	22 (15.9)	22 (15.9)
ypStage at enrollment		
Stage IIB	41 (29.7)	37 (26.8)
Stage IIIA	42 (30.4)	52 (37.7)
Stage IIIB	20 (14.5)	20 (14.5)
Stage IIIC	13 (9.4)	7 (5.1)
Stage IV	22 (15.9)	22 (15.9)
Number of lymph node metastasis		
pN1 (1–2)	78 (56.5)	80 (58.0)
pN2 (3–6)	44 (31.9)	43 (31.2)
pN3 (≥ 7)	16 (11.6)	15 (10.9)
ECOG PS at baseline^a^		
0	100 (72.5)	102 (73.9)
1	38 (27.5)	36 (26.1)

*N* = Total number of participants in the relevant analysis; *n* = Number of participants in each category. % = Percentage of participants in each category relative to the total number of participants in the relevant analysis population. Data are presented as *n* (%) unless otherwise stated

*ECOG PS* Eastern Cooperative Oncology Group performance status, *DCF* docetaxel + cisplatin + 5-fluorouracil, *FP* 5-fluorouracil + cisplatin, *FAP* adriamycin + 5-fluorouracil + cisplatin

^a^Baseline was defined as the last available assessment obtained between screening and study visit 1

**Fig. 1:**
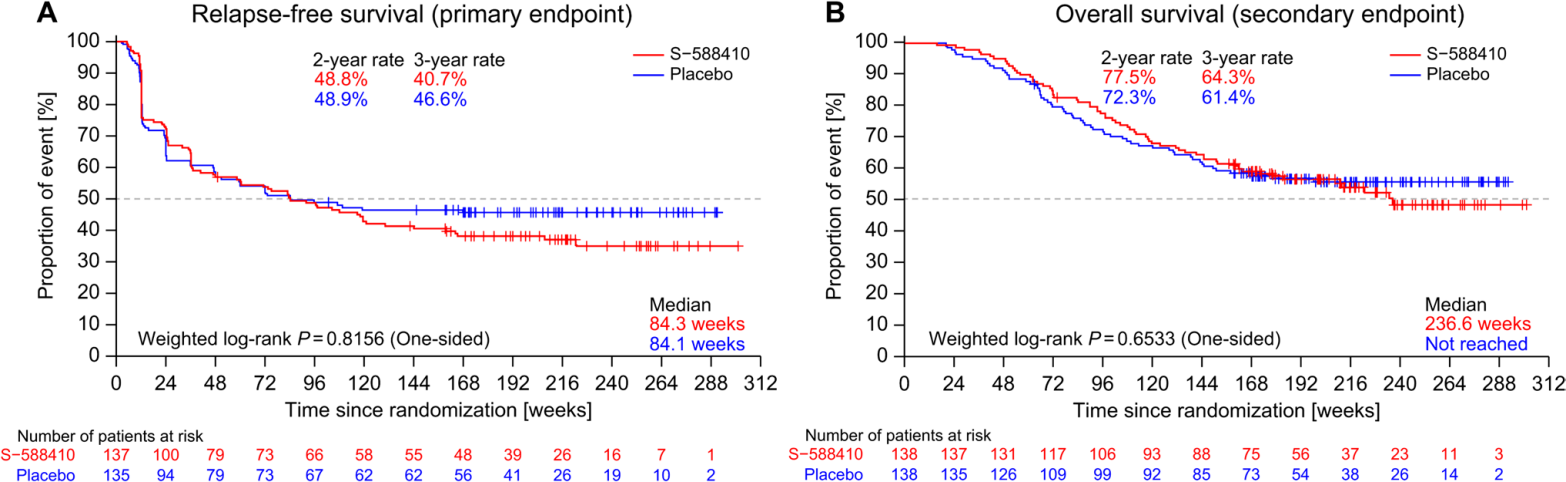
Kaplan–Meier estimates of relapse-free survival (**A**) and overall survival (**B**) in patients with ESCC treated with S-588410 and placebo. Weighted log-rank = weighted log-rank test with Fleming-Harrington class of weights for *ρ* = 0 and *γ* = 0.5

To evaluate the efficacy, RFS (primary endpoint) and OS (secondary endpoint) were estimated for the ITT population. The median RFS was 84.3 (90% CI 48.3–119.6) weeks and 84.1 weeks (48.1 weeks–not reached) in the S-588410 and placebo groups, respectively (Fig. 1A). The median OS was 236.3 weeks (187.7 weeks–not reached) and not reached (177.6 weeks–not reached) in the S-588410 and placebo groups, respectively (Fig. 1B). The median DFS (a secondary endpoint) was 83.3 (90% CI 47.1–118.1) weeks and 72.1 (47.1–163.9) weeks in the S-588410 and placebo groups, respectively. There were no significant differences in RFS (weighted log-rank test; one-sided *P* = 0.8156), OS (*P* = 0.6533), or DFS (weighted log-rank test; two-sided *P* = 0.3621) between the groups.

To evaluate immune responses in PBMCs during cancer vaccination, peptide-specific spots were measured using the ELISPOT assay. In the S-588410 group, the CTL induction rate against any of the five antigens at least once during the first 12 weeks of treatment was 98.5% (132/134 evaluated in the ITT population), with induction rates of 93.3%, 53.7%, 29.9%, 63.2%, and 65.7% for URLC10 (125/134), CDCA1 (72/134), KOC1 (40/134), DEPDC1 (84/133), and MPHOSPH1 (88/134) peptides, respectively. As shown in Online Resource 7A (Fig.), the highest proportion of participants (28.4%, 38/134) showing CTL induction within 12 weeks was attributed to the group that was positive for the three antigens. URLC10, DEPDC1, and MPHOSPH1 generated strong CTL induction (3+), whereas CDCA1 and KOC1 were associated with poor CTL induction (Fig., Online Resource 7B). Given the relatively low CTL induction rate of KOC1, the number of spots for the other four peptides is represented as scatter plots to evaluate the relationship between the two peptides in all combination patterns (Fig., Online Resource 7C). The number of specific spots (mean ± standard deviation) was 209 ± 232 for URLC10, 25 ± 67 for CDCA1, 51 ± 110 for DEPDC1, and 60 ± 114 for MPHOSPH1. Among the four spots, CDCA1 had a low mean number of spots, and patients with ≥ 100 specific spots (high CTL intensity) against CDCA1 had a relatively low number of specific spots against MPHOSPH1, and vice versa (Fig., Online Resource 7C).

As shown in the figure in Online Resource 4, 140 and 136 participants in the S-588410 and placebo groups, respectively, were included in the safety analysis. Treatment-related adverse events (TRAEs) occurred in 137 of 140 patients (97.9%) in the S-588410 group (239 events), and in 116 of 136 patients (85.3%) in the placebo group (228 events). Deaths during the treatment period occurred in two patients in the S-588410 group (one death due to malnutrition and aspiration pneumonia, one death due to myocardial infarction) and one patient in the placebo group (one death due to a malignant neoplasm of unknown primary site). All events were determined to be unrelated to the study drug. Table 2 summarizes TRAEs with an incidence of ≥ 1%. In both groups, the most frequent TRAE was injection site reaction. Grade 3 injection-site reactions (12.9%, 18/140 in the safety analysis population) were observed only in the S-588410 group. Other grade 3 TRAEs included injection-site infection, reduced neutrophil count, and reduced white blood cell count in the S-588410 group (0.7% each in 1/140) and hypokalemia in the placebo group (0.7% each in 1/136). TRAEs resulting in treatment withdrawal included eczema and elevated eosinophil counts in the S-588410 group (0.7% each in 1/140) and nausea and malaise in the placebo group (0.7% each in 1/136). No grade 4 or 5 TRAEs were reported.

**Table 2:** TRAEs in the S-588410 and placebo groups (incidence ≥ 1% each in the group, safety analysis population)

	S-588410 *N* = 140, *n* (E) %	Placebo *N* = 136, *n* (E) %
Any	137 (239) 97.9	116 (228) 85.3
Injection-site reaction	137 (177) 97.9	113 (208) 83.1
Pyrexia	12 (28) 8.6	1 (1) 0.7
Injection-site infection	3 (3) 2.1	0
Malaise	2 (2) 1.4	2 (2) 1.5
Hypersensitivity	2 (14) 1.4	0
Rash	2 (2) 1.4	1 (1) 0.7
Gynecomastia	2 (2) 1.4	0

*N* = Total number of participants in the relevant analysis population. *n* = Number of participants with an adverse event of that type; each participant was counted only once. % = Percentage of participants in the relevant analysis population who experienced an adverse event of that type. *E* = Number of adverse events of that type; that is, a participant experienced multiple adverse events counted multiple times

Treatment-emergent adverse events were classified by system organ class and preferred terms, using the Medical Dictionary for Regulatory Activities (MedDRA) Version 23.0

*TRAE* treatment-related adverse event

Based on subgroup analysis by tumor lesion, S-588410-treated participants had significantly longer survival than placebo-treated participants with upper thoracic ESCC (Fig., Online Resource 8). In the subgroup analysis based on stratification factors, no significantly prolonged survival was observed in the S-588410 group according to the histopathological classification of lymph node metastasis (pN1, pN2, or pN3) and classification of neoadjuvant therapy (Figs., Online Resource 9 and 10). Analysis of histopathological and clinical TNM classification and stage revealed that the S-588410 group was not associated with significantly prolonged survival compared to the placebo group (data not shown). As an exploratory analysis, we examined the relationship between the CTL intensity and survival. Patients with ≥ 100 peptide-specific spots (high CTL intensity population in Online Resource 7C) for each CDCA1 and MPHOSPH1 peptide tended to exhibit prolonged RFS and OS (Fig., Online Resource 11A, B) in the late phase, although the difference was not significant. Analysis of KOC1 was not performed because none of the patients had ≥ 100 peptide-specific spots. Similar results were obtained when analyzing patients with a positive CTL induction or those with a 3 + CTL grade (data not shown). In the S-588410 group, participants (*n* = 18) with grade 3 injection-site reactions had a longer RFS and OS than those with grade < 3 reactions (Fig., Online Resource 11C, D).

## Discussion

To the best of our knowledge, this phase 3 study is the first to investigate the efficacy, immune response, and safety of the multi-peptide cancer vaccine S-588410 as an adjuvant monotherapy after curative resection in patients with ESCC. No statistically significant differences were observed in RFS or OS between the S-588410 and placebo groups. Conversely, prolonged survival was observed in a subgroup of patients with upper thoracic ESCC, grade 3 injection-site reactions, and a high immune response, with ≥ 100 peptide-specific spots for CDCA1 and MPHOSPH1.

Regarding immune response, the CTL induction rate of each peptide in S-588410 in the current phase 3 study was similar to levels achieved in the phase 2 bladder cancer study of S-588410 [17]. However, compared with the results of the phase 2 esophageal cancer study using three peptides (URLC10, CDCA1, and KOC1), despite the similar CTL induction rates for URLC10 (93.3% [125/134] in the phase 3 study and 87.9% [29/33] in the phase 2 study), induction rates of CDCA1 and KOC1 in the current study were lower than those in the phase 2 study (53.7% [72/134] in the phase 3 study and 90.9% [30/33] in the phase 2 study for CDCA1; 29.9% [40/134] in the phase 3 study and 54.5% [18/33] in the phase 2 study for KOC1) [10]. Considering the grade of CTL response, the proportion of patients with a high grade (3+) for CDCA1 was lower in the phase 3 study than in the phase 2 ESCC study [10]. Treatment with a cancer peptide vaccine has been associated with an augmented CTL induction (as an immune response) and survival benefits [9–11, 18–24]. These results suggest that a high CTL response for CDCA1 critically affects survival efficacy, although differences in study design, such as the control group and number of administered peptides between the phase 2 ESCC study [10] and current phase 3 study, need to be considered.

Given that the number of peptide-specific spots could be employed as an evaluation criterion for the immune response [24, 25], we measured the difference in survival between patients with ≥ 100 peptide-specific spots and those with < 100 spots for each peptide. Reportedly, patients with > 100 spots for TTK, LY6K (also known as URLC10), and IMP-3 (also known as KOC1) post-vaccination exhibit clinical response [25]. Although we failed to detect statistical significance in our study, patients with ≥ 100 peptide-specific spots for CDCA1 and MPHOSPH1 tended to exhibit prolonged survival. However, limited reports are available regarding the criteria for the number of peptide-specific spots, thus, it may be necessary to evaluate this number as a novel indicator. Furthermore, patients with a high number of specific spots for both CDCA1 and MPHOSPH1 were rarely detected, although some patients exhibited numerous spots for the other peptide pairs (Fig., Online Resource 7C). The precise reason underlying the limited number of patients exhibiting high numbers of specific spots for both CDCA1 and MPHOSPH1 remains unknown, despite peptides from CDCA1 and MPHOSPH1 being categorized as “strong” epitopes according to peptide–HLA-binding prediction by NetMHC-4.0 [26, 27]. Further investigations are warranted to determine whether competition among co-administered peptides could explain the restricted CTL induction. In the presence of such competition, appropriate selection of a combination of peptides may be essential for mixed-peptide cancer vaccines. In some studies, each peptide was injected separately as a multipeptide vaccine [9, 19]. However, a previous study found no significant difference in immune response induction between multiple-site injections of each peptide and single-site injections of a cocktail peptide [18]. Although the CDCA1 peptide has been explored in other clinical studies [19–22], S-588410 is the first cocktail vaccine in which CDCA1 was mixed with MPHOSPH1. Thus, before initiating a pivotal study, it is necessary to establish whether the CTL response level for each peptide is reproducible compared with that observed in previous studies, especially when considering new multipeptide formulations.

In a pre-surgical study conducted on esophageal cancer, increased expression of PD-L1 was observed in tumors following administration of a cancer vaccine [16]. In patients in whom a cancer vaccine alone is ineffective, the induction of PD-L1 expression is thought to be the cause of the reduced efficacy, so the combination of a cancer vaccine with a PD-(L)1 antibody is expected to be effective. In particular, a synergistic effect may be seen in patients whose tumor immunity is not very active. In addition, S-588410 administration in a pre-surgical study on esophageal cancer has also shown to induce functional CD8 + and CD4 + TILs, specifically CD8 + PD-1 + and CD4 + PD-1 + cells [16]. This induction of TILs may be associated with an increase in interferon-gamma production. Consequently, it is possible that this increased interferon-gamma production from TILs may lead to an upregulation of PD-L1 expression in the tumor tissue, which allows cancer cells to evade immune detection. Checkpoint inhibitors, such as pembrolizumab or nivolumab, can block the PD-L1/PD-1 interaction, reactivating the immune response against cancer cells. Thus, combining S-588410 administration with checkpoint inhibitors may further enhance the efficacy of these immunotherapies in esophageal cancer treatment. Based on the above, it is expected that the combination with the standard therapy, PD-1 antibody, will be clinically significant.

Although various phase 3 trials have reported the efficacy of cancer peptide vaccines [28–31], factors associated with survival remain poorly established. In this trial, patient demographics, and biomarkers, including CTL responses, were evaluated in patients; however, none of these were associated with survival. In contrast, we observed a substantial survival benefit afforded by S-588410 in patients with upper thoracic ESCC. Although the mechanism of this benefit requires further investigation, our findings might be valuable as a possible treatment option since it has been reported that upper thoracic ESCC had worse survival than those with mid- and lower thoracic ESCC [32] and no consensus for the optimal treatment of upper thoracic ESCC has been provided [3]. Moreover, we observed that patients with grade 3 injection-site reactions in the S-588410 group showed significantly prolonged survival, consistent with the findings of previous cancer vaccine trials [18, 19, 23, 28]. Thus, a strong local immune reaction at the injection site could be a prognostic factor for cancer vaccine treatment. One possible mechanism of action of cancer peptide vaccines is induction of potent immune responses. Accordingly, it is crucial to develop a method to reinforce CTL induction and/or to select patients for whom cancer peptide vaccines are immunologically beneficial.

Nevertheless, this study had some limitations. First, the S-588410 comprises only HLA-A*24:02-restricted peptides. Therefore, we speculate that our findings are limited to HLA-A*24:02-positive individuals (most common in the Japanese population) owing to the use of HLA-A*24:02-restricted peptides. Second, validating the analysis of the number of peptide-specific spots remains a challenge, given the few patients with high CTL intensity and limited number of comparable reports.

In conclusion, although the current study failed to meet its primary endpoint, our findings revealed that S-588410 may confer a survival benefit to patients with ESCC capable of developing an immune response upon vaccination with certain key peptides. Further studies are required to define the subset of patients with a high probability of developing an appropriate CTL response.

## Supplementary Information

Below is the link to the electronic supplementary material.

Supplementary file1 (DOCX 1937 KB)

## Data Availability

The data obtained in this study are not available in a public repository, because Shionogi & Co., Ltd. takes suitable measures to protect personal information and the sponsor’s intellectual property. The protected information is tailored to a specific request.
