# Supplementary material for: A phase 3, randomized, double-blind, multicenter, placebo-controlled study of S-588410, a five-peptide cancer vaccine as an adjuvant therapy after curative resection in patients with esophageal squamous cell carcinoma
Source: Esophagus. 2024 Jul 11;21(4):447–55. doi: 10.1007/s10388-024-01072-w (PMC11405444; doi:10.1007/s10388-024-01072-w)

**Article Title: A phase 3, randomized, double-blind, multicenter, placebo-controlled study of S-588410, a five-peptide cancer vaccine as an adjuvant therapy after curative resection in patients with esophageal squamous cell carcinoma**

**Journal Name:** Esophagus

**Author Name:** Tomoki Makino MD, PhD^1^; Hiroshi Miyata MD, PhD^2^; Takushi Yasuda MD, PhD^3^*; Yuko Kitagawa MD, PhD^4^; Kei Muro MD, PhD^5^; Jae-Hyun Park PhD^6^; Tetsuro Hikichi MHSc^7^; Takahiro Hasegawa DPH^8^; Kenji Igarashi MSc^9^; Motofumi Iguchi MSc^10^; Yasuhide Masaoka MSc^11^; Masahiko Yano MD, PhD^2^**; and Yuichiro Doki MD, PhD^1^

**Affiliation:**

^1^Department of Surgery, Gastroenterological Surgery, Graduate School of Medicine, Osaka University, Osaka, Japan

^2^Department of Gastroenterological Surgery, Osaka International Cancer Institute, Osaka, Japan

^3^Department of Surgery, Kindai University Faculty of Medicine, Osaka, Japan

^4^Department of Surgery, Keio University School of Medicine, Tokyo, Japan

^5^Department of Clinical Oncology, Aichi Cancer Center Hospital, Nagoya, Aichi, Japan

^6^OncoTherapy Science, Inc., Kawasaki, Kanagawa, Japan

^7^Laboratory Department, Cancer Precision Medicine, Inc., Kawasaki, Kanagawa, Japan

^8^Biostatistics Center, Shionogi & Co., Ltd., Osaka, Japan

^9^Project Management, Shionogi & Co., Ltd., Osaka, Japan

^10^Medical Affairs Department, Shionogi & Co., Ltd., Osaka, Japan

^11^Clinical Development, Shionogi & Co., Ltd., Osaka, Japan

**Current institution: Suita Municipal Hospital, Osaka, Japan

*Corresponding author:

Prof. Takushi Yasuda

Department of Surgery, Kindai University Faculty of Medicine

E-mail: [takushi-yasuda6008@med.kindai.ac.jp](mailto:takushi-yasuda6008@med.kindai.ac.jp)

**Online Resource 1:** Study design.





**Online Resource 2**

*Inclusion criteria*

Participants were eligible of they met the selection criteria listed below.

When obtaining consent

1) Patients whose primary tumor site was in the thoracic esophagus according to UICC 7th edition.

2) Patients who received chemotherapy including platinum agents and fluoropyrimidine anticancer drugs as a neoadjuvant chemotherapy (radiotherapy).

3) Patients who underwent esophagectomy by radical surgery for esophageal cancer classified as R0 (histopathologically no residual cancer) by residual tumor of UICC 7th edition.

4) Patients who underwent lymph node dissection and had histopathologically confirmed positive lymph node metastasis.

5) Patients with histologically confirmed esophageal squamous cell carcinoma (ESCC).

6) Male or female patients aged ≥ 20 years.

7) Patients who provided written consent to participate in the clinical trial.

At the time of registration

8) Patients with histopathological Stage IIB, Stage III, Stage IV (M1 cervical lymph node metastasis only) according to UICC 7th edition.

9) Patients with HLA-A allele HLA-A*24:02.

10) Patients with East Coast Cancer Group (ECOG) performance status (PS) 0 or 1.

11) Patients who were able to receive the investigational drug within 14–56 days after surgery.

12) Patients who are willing and able to provide tumor tissue for confirmation of antigen expression. The submitted specimen was lumin-fixed in paraffin-embedded blocks or sections.

*Exclusion criteria*

Patients who met any of the following criteria at the time of registration were excluded.

1) Patients with active multiple cancers (including a history of multiple cancers with a disease-free interval of 5 years or less). However, lesions equivalent to intraepithelial carcinoma or intramucosal carcinoma that have been treated endoscopically and were deemed to be cured were not included in active multiple cancers.

2) Patients who received other postoperative adjuvant therapies (chemotherapy, radiation therapy, immunotherapy, hyperthermia therapy, and other treatments effective in preventing recurrence).

3) Patients for whom 21 days or more have not passed since the end of the following treatment or administration (last treatment date or last administration date):

-Anti-neoplastic drugs

-Radiotherapy

-Heat therapy

-Systemic* corticosteroids (corticosteroids with a prednisolone equivalent of 10 mg or less)

(excluding oral administration)

-Systemic*immunosuppressants

-Immunotherapy

-Chinese herbal medicines with anticancer effects or systemic* immune effects

*: Systemic is defined as use for purposes other than local action (transdermal, ear drops, eye drops, inhalation, nasal spray).

4) Patients with serious liver damage, renal damage, or cardiac damage respiratory disease, metabolic disease, etc. of grade 3 or higher according to Common Terminology Criteria for Adverse Events (CTCAE) version 4.03.

5) Patients who were scheduled to be treated with concomitant drugs/concomitant use prohibited during the period from registration to the end of the follow-up period (or discontinuation of the treatment period/post-observation period).

6) Patients whose test values of bone marrow, liver, and renal function confirmed by clinical examination 14 days after radical resection of esophageal cancer and within 28 days before enrollment meet the following criteria.

-White blood cell count < 2000/mm^3^, > 15000/mm^3^

-Hemoglobin < 8.0 g/dL

-Platelet count < 50000/mm^3^

-Total bilirubin > 2.0 mg/dL

-Aspartate aminotransferase (AST) or alanine aminotransferase (ALT) > 150 U/L

-Serum creatinine > 3.0 mg/dL

7) Patients with increased eosinophil count (> 5000/mm^3^) within 28 days before registration, and patients with eosinophilic pneumonia or a history of interstitial pneumonia or complications.

If symptoms and findings of pneumonia were suspected before registration, the investigator (sub-investigator) performed additional tests such as chest X-rays to detect eosinophilic pneumonia or check for complications of interstitial pneumonia.

8) Patients with known human immunodeficiency virus (HIV) infection.

9) Patients with systemic or active infection that is difficult to control.

10) Patients who were at risk of sudden death due to experiencing the following within 12 months of registration.

-Coronary artery or peripheral artery bypass grafting

-Transient ischemic attack

-Symptoms due to myocardial infarction or unstable angina pectoris

-Symptoms due to pulmonary embolism or deep vein thrombosis.

11) Patients with complications, history, or symptoms of autoimmune disease or immunodeficiency disease.

[Examples]

-Collagen diseases (systemic lupus erythematosus, systemic scleroderma, etc.)

-Multiple sclerosis

-Rheumatoid arthritis

12) Patients with a history of serious allergic reactions due to administration of drugs, vaccines, or biological products (CTCAE version 4.03.

13) Patients who were hospitalized for treatment at the time of registration.

14) Female patients who were pregnant or breastfeeding. Female patients with a positive pregnancy test at enrollment. However, the following women who have no possibility of pregnancy were exempted from the test.

-Women who were menopausal (have not menstruated for more than 2 years without any other medical reason)

-Women who underwent surgical procedures that make them unable to conceive (hysterectomy, bilateral oophorectomy, tubal ligation)

15) Patients who do not intend to use the following contraceptive methods or who cannot use them from the start of study drug administration to 3 months after the final administration,

-Abstinence

-Oral contraceptives

-Intrauterine contraceptive devices

-Tubal ligation

-Vasectomy for male partner.

16) Patients who have ever received DEPDC1 peptide vaccine, MPHOSPH1 peptide vaccine, URLC10 peptide vaccine, CDCA1 peptide vaccine, or KOC1 peptide vaccine in the past.

17) Patients administered another investigational drug within 28 days or 5 times the half-life (whichever is longer) prior to registration.

18) Patients who are judged to be unsuitable for this trial by the investigator (co-investigator).

### Immunohistochemistry

Resected tumor tissues were subjected to immunohistochemistry staining to measure URLC10, CDCA1, KOC1, DEPDC1, and MPHOSPH1 expression levels during surgery. The tumor region was confirmed using hematoxylin and eosin staining. The five antigens were detected using antigen-specific antibodies and scored as previously described [1, 2]. The unavailability of sufficient specimens was recorded as “no results.”

Reference

[1] Daiko H, Marafioti T, Fujiwara T, et al. Exploratory open-label clinical study to determine the S-588410 cancer peptide vaccine-induced tumor-infiltrating lymphocytes and changes in the tumor microenvironment in esophageal cancer patients. Cancer Immunol Immunother. 2020;69:2247–2257.

## [2] Shimizu N, Hussain SA, Obara W, et al. A phase 2 study of S-588410 maintenance monotherapy for platinum-treated advanced or metastatic urothelial carcinoma. *Bladder Cancer*. 2022;8:179–192.

**Online Resource 3**

(1) Assumptions used to calculate the required sample size

| Item | Assumption |
| --- | --- |
| 3-year RFS rate in the placebo group | 30% |
| 3-year RFS rate in the S-588410 group | 45% |
| Time of onset of efficacy | 5 months |
| Long-term relapse-free rate in the placebo group | 25% |
| Long-term relapse-free rate in the S-588410 group | 40% |
| Time of relapse | At least 2 months after the start of follow-up |
| Patient accrual rate | Constant |
| Enrollment period | 2 years |
| Follow-up period after the end of enrollment | 2 years |

RFS, relapse-free survival

(2) Analysis of cytotoxic T-lymphocyte (CTL) induction rate

For the intention-to-treat (ITT) population, the CTL induction rates for the S-588410 group at weeks 8, 12, 32, 48, 96, and 108 of treatment and their 90% confidence intervals were calculated. The Clopper–Pearson method was used to calculate the confidence interval of the induction rate. In addition, the number of antigens that induced CTLs and the CTL induction rate by antigen were calculated for each time point evaluated. The presence or absence of CTL inducibility was determined relative to the baseline level. CTLs were regarded as “Induced” if the CTL activity increased by at least one level from baseline level at any time point after administering the study drug up to each time point of evaluation; otherwise, they were regarded as “Not induced”. Thus, if a patient discontinued study participation before protocol-specified evaluation time points, but the CTL induction assessment was performed at discontinuation, the result of this assessment would be used in the CTL induction analysis for the evaluation time points after discontinuation. The baseline level was defined as the CTL activity level before the first dose. The analysis population for evaluating the CTL induction rate and the number of antigens that induced CTLs at the time points of evaluation comprised patients for whom the presence or absence of CTL induction was determined and would be the same across different evaluation time points. Thus, this analysis excluded patients whose post-treatment CTL induction assessments were all NA, as well as patients whose baseline CTL assay results for the five antigens were all NA or 3+. Furthermore, the analysis population for the CTL induction rate by antigen excluded patients whose post-treatment CTL induction assessments for each antigen were all NA, as well as those patients whose baseline CTL assay results for each antigen were all NA or 3+.

(3) Analysis of the relationship between survival and CTL induction

The original analysis plan described the relationships between relapse-free survival (RFS)/overall survival (OS) and the presence or absence of CTL induction or the number of antigens that induced CTLs. A landmark analysis with the landmark at weeks 8, 12, 32, 48, 96, and 108 of treatment was performed using the Kaplan–Meier method, and a stratified log-rank test was used to assess the difference in RFS/OS. Additionally, differences in RFS/OS between the CTL intensity with < 100 and ≥ 100 peptide-specific spots were assessed using a stratified Cox proportional hazards model, with < 100 and ≥ 100 peptide-specific spots as a time-dependent covariate.

(4) Interim analysis

For the primary endpoint, the independent data monitoring committee (IDMC) decided to conduct an interim analysis according to the procedure shown in the following figure based on the number of relapse events (including deaths) observed in the ITT population under blinded conditions at 9 months after the end of enrollment.

For the ITT population, the superiority of S-588410 over the placebo in terms of the primary endpoint of RFS was assessed using the stratified log-rank test with Fleming–Harrington class of weights for (ρ, γ)=(0, 0.5). If the interim analysis showed a significant difference, the efficacy of the study drug would be declared, and the study would be terminated early for efficacy. To maintain the probability of type 1 error at 5.0% for the entire study, the multiplicity of tests in the interim and final analyses were adjusted using the Haybittle–Peto method. A one-sided significance level of 0.3% was used in the interim analysis. The significance level for the final analysis was calculated based on the information at the time of interim analysis which was defined as “(amount of information at the interim analysis)/(assumed amount of information at the final analysis)”. The assumed amount of information at the time of the final analysis was 12.43. The amount of information at the interim analysis was calculated under blinded conditions as follows [3].

| Amount of information at the interim analysis    *T_k_* (*k* = 1,.., *D*) is the time point at which a relapse event occurs before the interim analysis and *d_k_* is the number of relapse events in the combined group at time *T_k_*. The weighted log-rank test with the Fleming-Harrington class of weights for (*ρ*, *γ*) = (0, 0.5) are , where  is the RFS rate estimated using the Kaplan-Meier method in the combined group at time *T_k_*, and *D* is the number of different time points at which relapse events occurred by the time of interim analysis. |
| --- |

In contrast, if the stratified log-rank test with the Fleming–Harrington class of weights for (ρ, γ)=(0, 0.5) for RFS showed a one-sided *P* > 0.5, the study would be terminated for futility. If the interim analysis did not result in early stopping for efficacy or futility, the study would be continued.

Reference

[3] Hasegawa T. Group sequential monitoring based on the weighted log-rank test statistic with the Fleming–Harrington class of weights in cancer vaccine studies. Pharm Stat. 2016;15:412–419.

**Online Resource 4:** Participants disposition and analysis population.


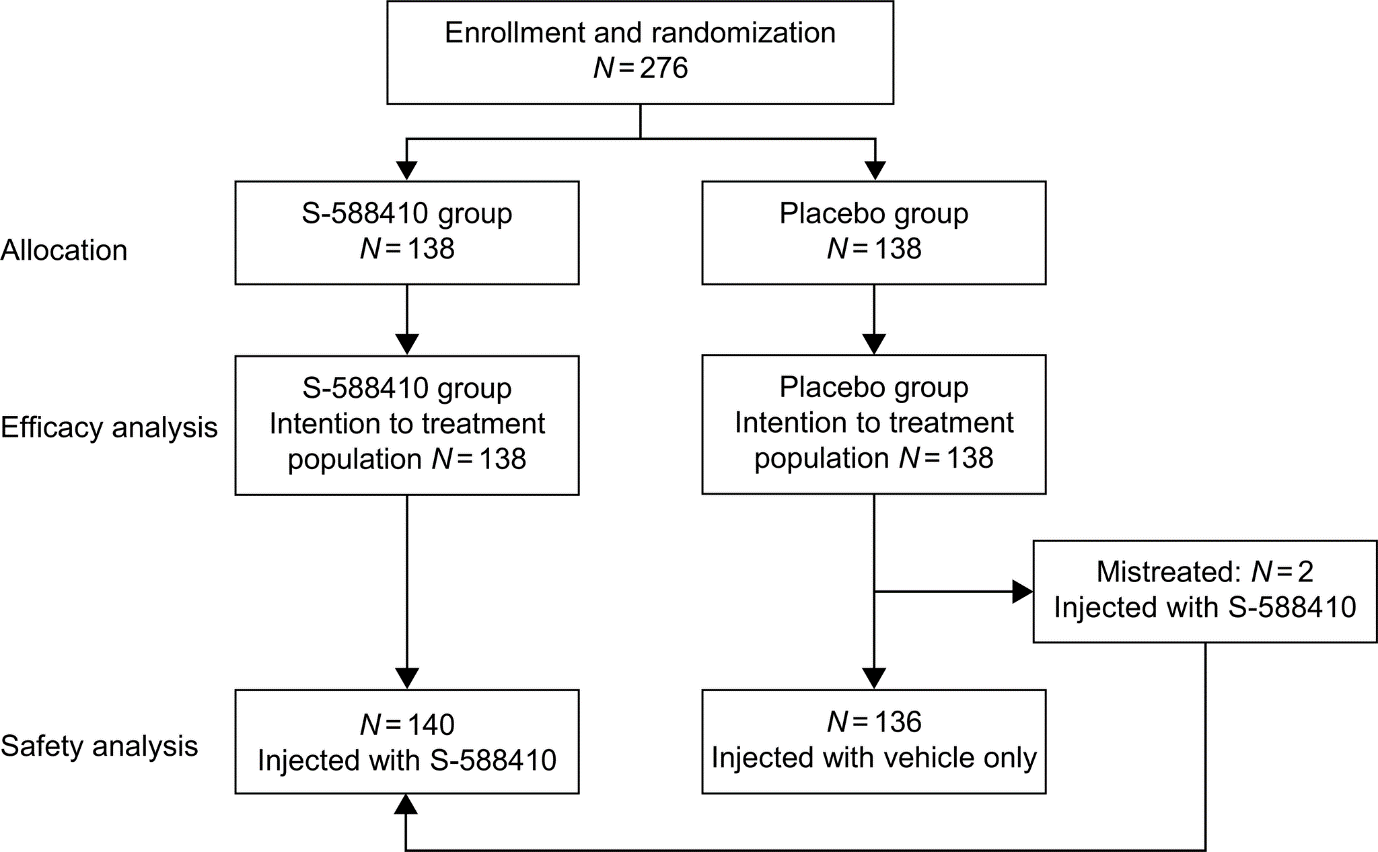


**Online Resource 5.** Clinical TNM classification and stage at diagnosis of participants in the intention-to-treat (ITT) population

|  | S-588410 *N*=138 | Placebo *N*=138 |
| --- | --- | --- |
| cTNM_T |  |  |
| T0 | 0 | 0 |
| T1 | 13 (9.4) | 11 (8.0) |
| T2 | 29 (21.0) | 31 (22.5) |
| T3 | 85 (61.6) | 85 (61.6) |
| T4 | 11 (8.0) | 11(8.0) |
| cTNM_N |  |  |
| N0 | 21 (15.2) | 13 (9.4) |
| N1 | 74 (53.6) | 78 (56.5) |
| N2 | 41 (29.7) | 42 (30.4) |
| N3 | 2 (1.4) | 5 (3.6) |
| cTNM_M |  |  |
| M0 | 117 (84.8) | 115 (83.3) |
| M1 | 21 (15.2) | 23 (16.7) |
| cStage |  |  |
| Stage IA | 0 | 1 (0.7) |
| Stage IB | 9 (6.5) | 4 (2.9) |
| Stage IIA | 10 (7.2) | 5 (3.6) |
| Stage IIB | 21 (15.2) | 27 (19.6) |
| Stage IIIA | 47 (34.1) | 39 (28.3) |
| Stage IIIB | 24 (17.4) | 28 (20.3) |
| Stage IIIC | 6 (4.3) | 11 (8.0) |
| Stage IV | 21 (15.2) | 23 (16.7) |

**Online Resource 6:** Percentage of patients by antigen expression in ESCC tissue at surgical resection in the intention-to-treat population. *N*=138/group.





**Online Resource 7:** Cytotoxic T-lymphocyte (CTL) response in PBMCs after 12 weeks of S-588410 vaccination. The proportion of participants by number of peptides (0–5) showing CTL induction to the total *n*=134 (A). Percentage of participants by CTL grades (negative, 1+, 2+, 3+) to the total *n*=127 for each peptide (B). Plots of the number of peptide-specific spots of *n*=127 (*n*=125 for MPHOSPH1) in all combination patterns of four peptides without KOC1 (C). The number of peptide-specific spots was calculated by subtracting baseline data from data at 12 weeks. CTL grades were determined using an algorithm for specific spots. CTL induction of each antigen was defined as increased corresponding CTL grade compared with baseline value at 8 or 12 weeks from the start of S-588410 vaccination.

Abbreviations: CDCA1, cell division cycle-associated 1; DEPDC1, DEP domain containing 1; KOC1, KH domain-containing protein overexpressed in cancer 1; MPHOSPH1, M-phase phosphoprotein 1; PBMC, peripheral blood mononuclear cell; URLC10, upregulated lung cancer 10


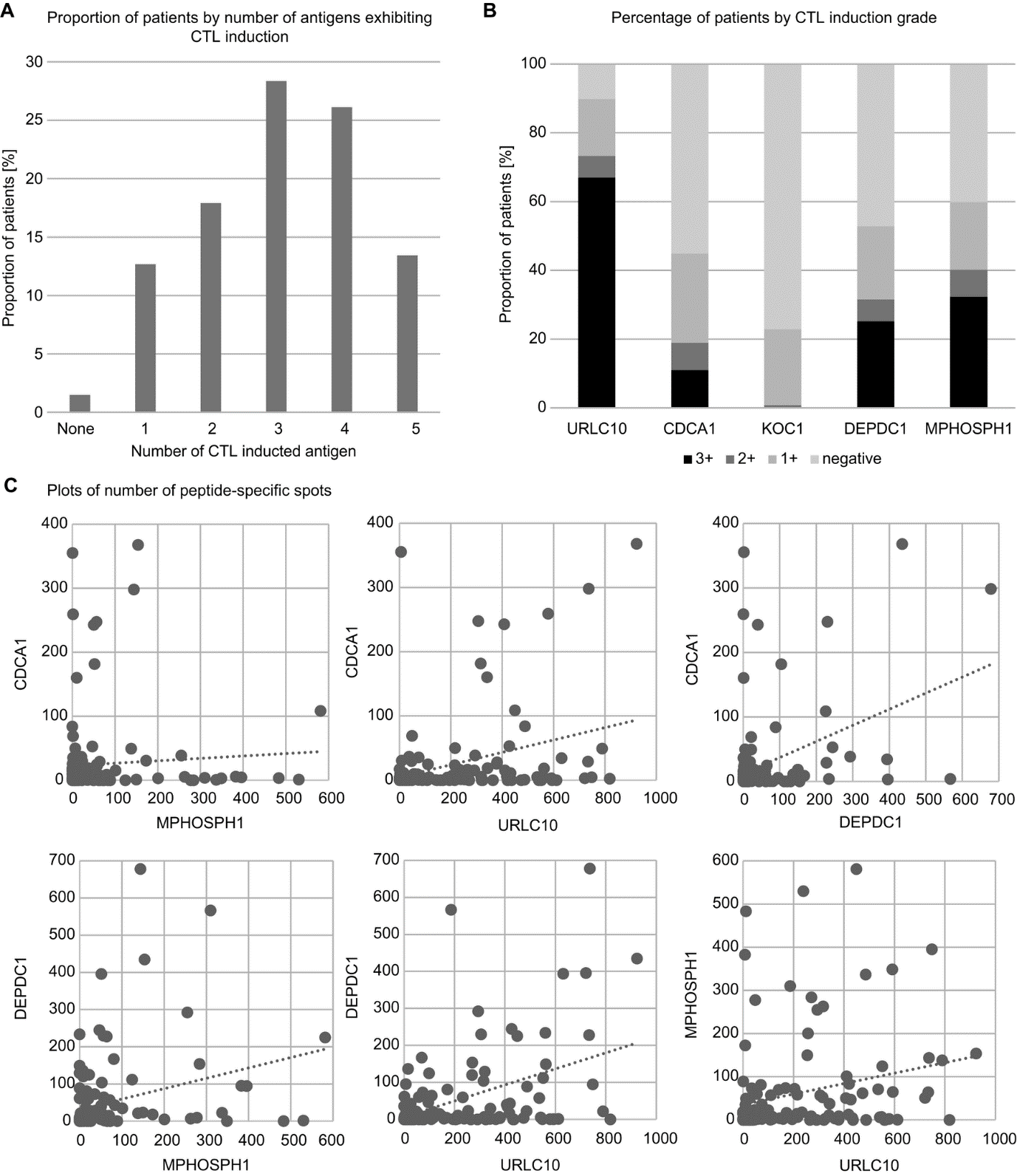


**Online Resource 8:** Kaplan–Meier estimates of relapse-free survival (A) and overall survival (B) by ESCC lesion in the intention-to-treat population who received S-588410 and placebo. Weighted log-rank=weighted log-rank test with Fleming–Harrington class of weights for ρ=0 and γ=0.5.


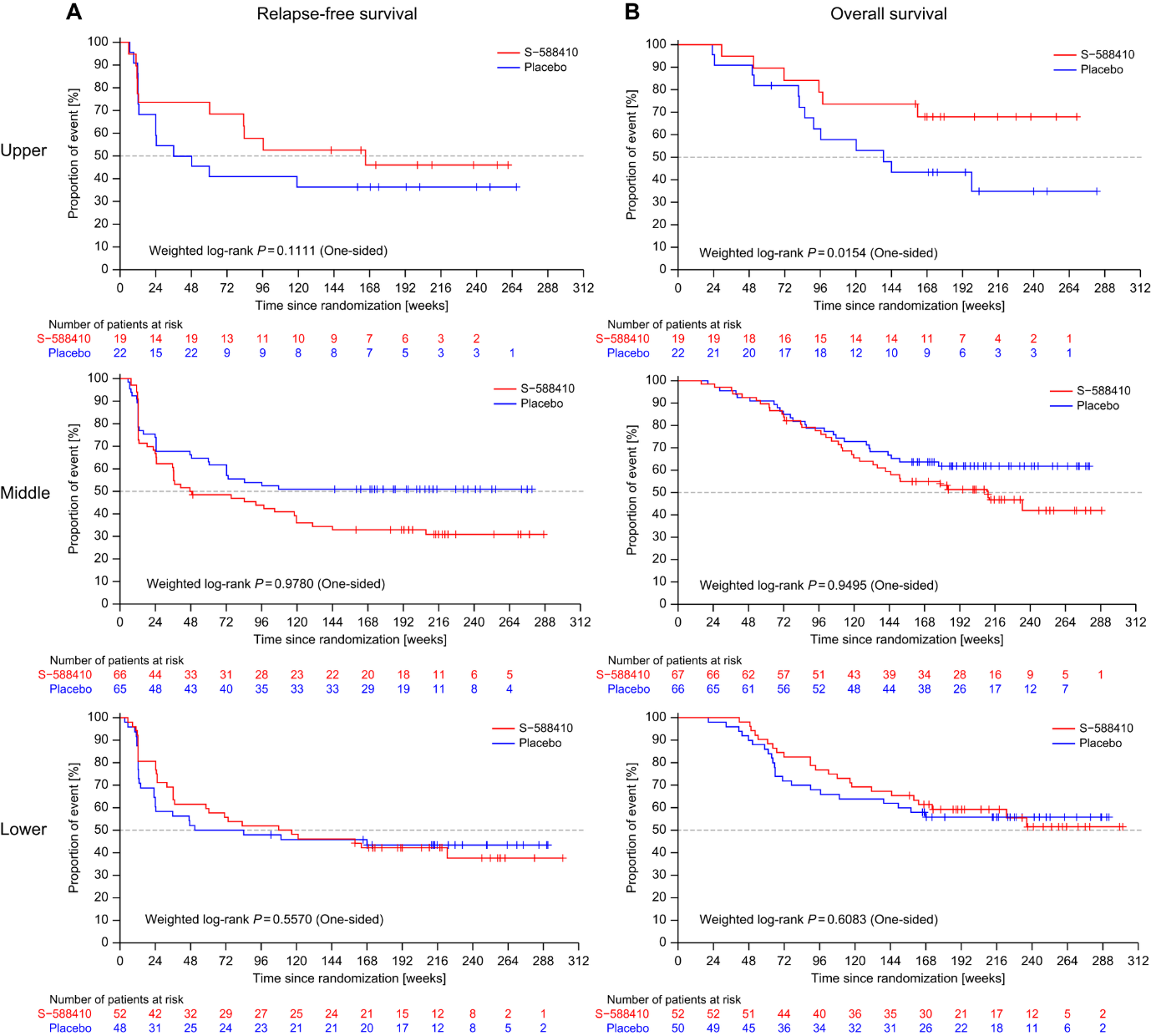


**Online Resource 9:** Kaplan–Meier estimates of relapse-free survival (A) and overall survival (B) by the histopathological classification of lymph node metastasis (pN1, pN2, or pN3) in the intention-to-treat population. Weighted log-rank=weighted log-rank test with Fleming–Harrington class of weights for ρ=0 and γ=0.5.


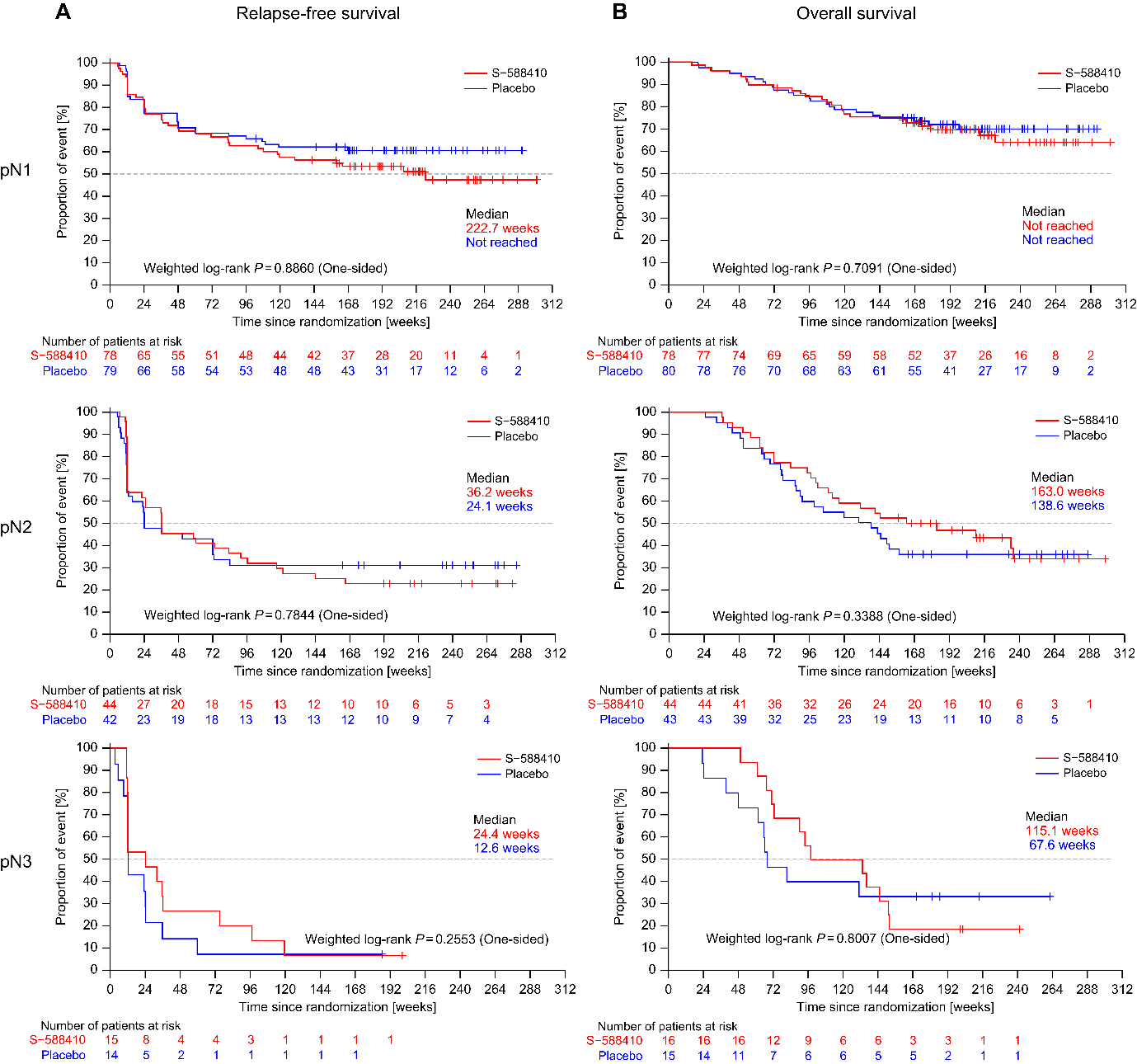


**Online Resource 10:** Kaplan–Meier estimates of relapse-free survival (A) and overall survival (B) by preoperative therapy in the intention-to-treat population. Weighted log-rank=weighted log-rank test with Fleming–Harrington class of weights for ρ=0 and γ=0.5.


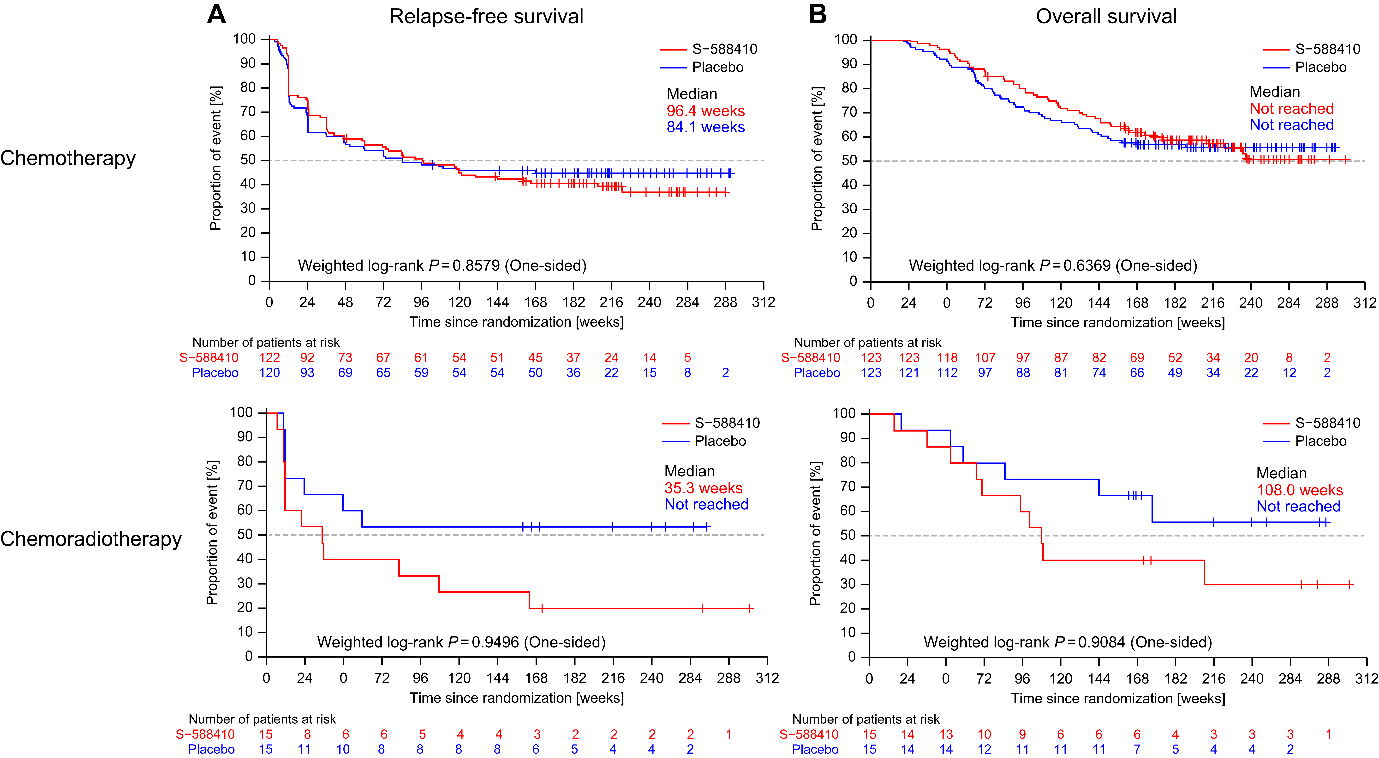


**Online Resource 11:** Kaplan-Meier estimates of relapse-free survival (A) and overall survival (B) in patients with a high number of peptide-specific spots and relapse-free survival (C) and overall survival (D) by the grade of injection-site reaction in the S-588410 group.


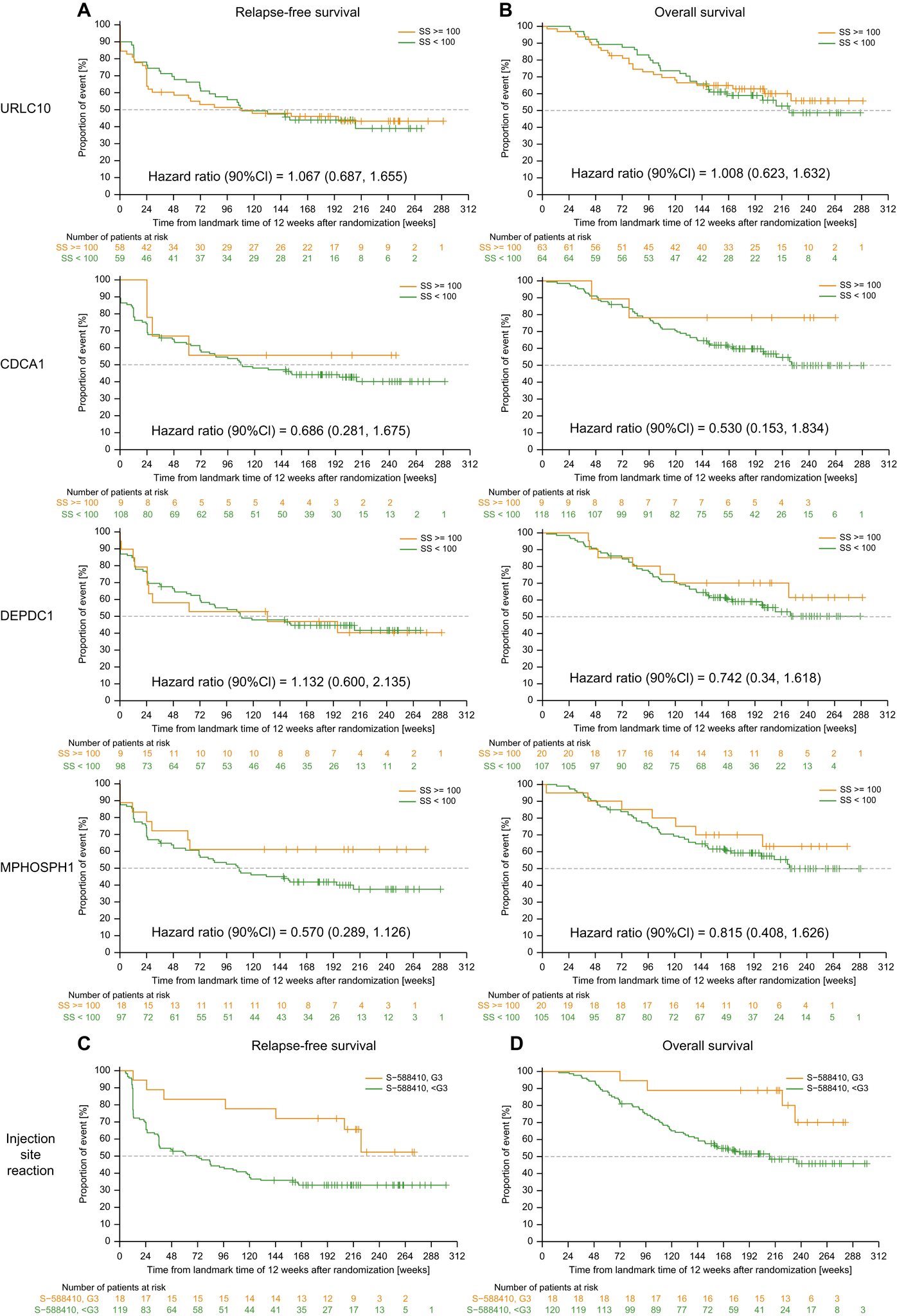

Supplement: Supplementary file 1 — Supplementary file1 (DOCX 1937 KB) [file 10388_2024_1072_MOESM1_ESM.docx]
